# Relaxin's induction of metalloproteinases is associated with the loss of collagen and glycosaminoglycans in synovial joint fibrocartilaginous explants

**DOI:** 10.1186/ar1451

**Published:** 2004-10-29

**Authors:** Tabassum Naqvi, Trang T Duong, Gihan Hashem, Momotoshi Shiga, Qin Zhang, Sunil Kapila

**Affiliations:** 1 Department of Orthodontics and Pediatric Dentistry, University of Michigan, Ann Arbor, Michigan, USA

**Keywords:** β-estradiol, collagen, collagenase-1, fibrocartilage, glycosaminoglycans, relaxin

## Abstract

Diseases of specific fibrocartilaginous joints are especially common in women of reproductive age, suggesting that female hormones contribute to their etiopathogenesis. Previously, we showed that relaxin dose-dependently induces matrix metalloproteinase (MMP) expression in isolated joint fibrocartilaginous cells. Here we determined the effects of relaxin with or without β-estradiol on the modulation of MMPs in joint fibrocartilaginous explants, and assessed the contribution of these proteinases to the loss of collagen and glycosaminoglycan (GAG) in this tissue. Fibrocartilaginous discs from temporomandibular joints of female rabbits were cultured in medium alone or in medium containing relaxin (0.1 ng/ml) or β-estradiol (20 ng/ml) or relaxin plus β-estradiol. Additional experiments were done in the presence of the MMP inhibitor GM6001 or its control analog. After 48 hours of culture, the medium was assayed for MMPs and the discs were analyzed for collagen and GAG concentrations. Relaxin and β-estradiol plus relaxin induced the MMPs collagenase-1 and stromelysin-1 in fibrocartilaginous explants – a finding similar to that which we observed in pubic symphysis fibrocartilage, but not in articular cartilage explants. The induction of these proteinases by relaxin or β-estradiol plus relaxin was accompanied by a loss of GAGs and collagen in joint fibrocartilage. None of the hormone treatments altered the synthesis of GAGs, suggesting that the loss of this matrix molecule probably resulted from increased matrix degradation. Indeed, fibrocartilaginous explants cultured in the presence of GM6001 showed an inhibition of relaxin-induced and β-estradiol plus relaxin-induced collagenase and stromelysin activities to control baseline levels that were accompanied by the maintenance of collagen or GAG content at control levels. These findings show for the first time that relaxin has degradative effects on non-reproductive synovial joint fibrocartilaginous tissue and provide evidence for a link between relaxin, MMPs, and matrix degradation.

## Introduction

In certain sites in and around joints, ligaments and tendons subjected to complex tensile and compressive loading specialize into fibrocartilaginous tissues [1-3] containing types I and II collagens and cartilage-specific proteoglycans. These tissues include specific regions of the metacarpophalangeal ligament and the deep flexor tendon, the temporomandibular joint (TMJ) disc, and the pubic symphysis. Within the pubic symphysis of several species, the reproductive hormone relaxin induces matrix remodeling activity during pregnancy and parturition, causing a marked decrease in collagen content through partly characterized mechanisms that transform this tissue into a ligamentous structure [4-9]. The relaxin-mediated loss of matrix macromolecules in the pubic symphysis and other tissues is exacerbated by estrogen [4,7,8,10]. The relative contribution of matrix synthesis and degradation to these relaxin-mediated changes is not clear, although collagen loss through increased proteolysis has been suggested [4], and studies in relaxin-knockout mice have implicated increased collagenase activity [11].

To understand the potential basis for relaxin and estrogen's modulation of the composition of fibrocartilaginous tissues, we previously studied cells isolated from rabbit TMJ discs. Relaxin induced the expression of the matrix metalloproteinases (MMPs) collagenase-1 (MMP-1) and stromelysin-1 (MMP-3) in a dose-dependent fashion but had little effect on the expression of tissue inhibitor of metalloproteinase-1 (TIMP-1) or TIMP-2 [12]. In cells primed with β-estradiol, however, the relaxin concentration required for maximal induction of collagenase-1 and stromelysin-1 was 90–99% lower than in unprimed cells. Notably, the MMP response to relaxin was specific to fibrocartilaginous cells and was not observed in TMJ synoviocytes. These findings suggest that relaxin, by targeting fibrocartilage, might predispose women to musculoskeletal diseases of fibrocartilaginous joints.

One such disease is TMJ disorders, which affect some 11 million adults in the USA [13,14], predominantly women, with a female : male ratio of 2:1 to 6:1 [14]. Unlike similar diseases of other joints, TMJ disorders occur primarily in women of reproductive age [14]. Given the gender and age distribution of these disorders and the relaxin-induced loss of matrix macromolecules in the pubic symphysis fibrocartilage [4,6,7,9] and isolated TMJ fibrocartilaginous cells [12], we have proposed that relaxin compromises the integrity of fibrocartilaginous tissues by enhancing the degradation of their matrices directly through the induction of specific MMPs. However, although relaxin causes a loss of collagens and proteoglycans in reproductive organs [6,7] and also increases MMP expression in specific tissues [6,12,15-21], the induction of MMPs by relaxin has not been demonstrated in joint fibrocartilaginous tissues or its induction of MMPs has not been linked to the loss of matrix macromolecules in any tissue.

In this study we determined the effects of relaxin with or without β-estradiol on the modulation of MMPs, and assessed the contribution of these proteinases to the changes in collagen and glycosaminoglycan (GAG) content in fibrocartilaginous disc explants. Our findings are consistent with the hypothesis that relaxin-mediated induction of MMPs is associated with the loss of matrix macromolecules that could compromise tissue function and biomechanics and might lead to joint disease.

## Materials and methods

### Materials

Twenty-week-old female New Zealand white rabbits were obtained from Nita Bell Laboratories (Hayward, California, USA). Ketamine hydrochloride was from Parke Davis (Morris Plains, New Jersey, USA), and xylazine was from Rugby Lab (Rockville Center, New York, USA). Lactalbumin hydrolysate, α-casein, β-estradiol-17-valerate, pepsin, papain, chondroitin sulfate A sodium from bovine trachea, Safranin-O, Fast Green, cetylpyridinium chloride, and other reagents were from Sigma (St Louis, Missouri, USA). 1,9-Dimethylmethylene blue (DMMB) was from Molecular Probes (Eugene, Oregon, USA), and ^35^S was from Amersham (Arlington Heights, IL, USA). Protein assay kits, gelatin (EIA grade), and nitrocellulose membrane were from Bio-Rad (Hercules, California). α-Minimal essential medium, trypsin, penicillin–streptomycin, and Fungizone^® ^were from Gibco (Grand Island, New York, USA). All other standard chemicals were from Sigma or Fisher Scientific (Pittsburg, Pennsylvania, USA).

Rabbit anti-human collagenase-1 polyclonal antibody and rabbit anti-mouse stromelysin-1 monoclonal antibody, horseradish peroxidase-conjugated secondary antibodies, and the MMP inhibitor GM6001 and its control analog were from Chemicon International (Temecula, California, USA). Rabbit anti-human-TIMP-1 antibody that cross-reacts with the rabbit inhibitor [12] was from Triple Point Biologics (Forest Grove, Oregon, USA). Enhanced chemiluminescence reagent for western blotting was from Amersham International (Little Chalfont, Bucks., UK). Sircol collagen assay kit was from Accurate Chemical and Scientific Corporation (Westbury, New York, USA), and fluorescein isothiocyanate (FITC)-labelled collagen was from Chondrex (Seattle, Washington, USA). Recombinant human relaxin was kindly provided by Connetics Corporation (Palo Alto, California, USA).

### Retrieval and culturing of TMJ discs, pubic symphysis, and articular cartilage

All procedures on rabbits were approved by the Committee on Animal Research of the University of California, San Francisco, and conducted in accord with accepted standards of humane animal care. Rabbits were anesthetized with ketamine hydrochloride (40 mg/kg) and xylazine (3–5 mg/kg), and the TMJ discs were harvested bilaterally under sterile conditions and immediately placed in calcium-free and magnesium-free phosphate-buffered saline (PBS) containing antibiotics (100 U/ml penicillin, 100 mg/ml streptomycin, and 100 U/ml Fungizone). After removal of the synovium under a dissecting microscope, each disc was washed three times in PBS and bisected longitudinally such that four samples from each rabbit were available (three for hormone treatments and one for control). The hemisections were weighed, placed in wells of a 24-well culture plate, covered with 1 ml of serum-free medium (phenol-free α-minimal essential medium with 0.2% lactalbumin hydrolysate, glutamine, nonessential amino acids, 100 U/ml penicillin, and 100 mg/ml streptomycin) with or without hormones, and cultured at 37°C in air containing 5% CO_2_.

For determination of MMPs and GAG staining, 32 hemisections from eight rabbits were exposed to medium alone, β-estradiol (20 ng/ml), relaxin (0.1 ng/ml), or both hormones at the same doses for 48 hours. The conditioned medium was collected and stored for MMP assays, and the discs were processed for GAG staining. To assess the contribution of relaxin-induced MMPs to the loss of collagen and GAGs, 24 hemisections from six rabbits were cultured with the MMP inhibitor GM6001 or its control analog 2 hours before and during the hormone treatments. The inhibitor was used at 10 μM, because this concentration was shown to inhibit collagenase activity induced by 0.1 ng/ml relaxin in dose–response experiments to baseline levels. The conditioned medium was collected and stored at -70°C for total protein and MMP assays. The discs were dried in a SpeedVac^®^, weighed, digested, and used for the determination of GAG and collagen content.

To determine whether the observed induction of collagenase by relaxin is specific to fibrocartilage, experiments were performed with pubic symphysis fibrocartilage, which is a known target site for β-estradiol and relaxin as a positive control, and with articular cartilage from the knee. For retrieval of articular cartilage, the joint was shaved, the articular surfaces were exposed, and the cartilage was scraped from the articular surfaces of the femur and tibia and incubated in PBS with antibiotic as described above. Similarly, the pubic bones and symphyseal areas were exposed under sterile conditions and the pubic symphysis (fibrocartilaginous tissues between the pubic bones) was dissected, removed, and incubated in PBS with antibiotics. The tissues were weighed, placed in wells of a 24-well culture plate, and studied as described above.

### Western blotting

Hormone-induced changes in collagenase-1, stromelysin-1, and TIMP-1 were determined by western blotting. Disc-conditioned medium was mixed with 4 × sample buffer and subjected to SDS–polyacrylamide-gel electrophoresis with 10% or 18% gels. Equal amounts of protein (determined with a bicinchoninic acid protein assay kit) were loaded in each lane. The proteins were transferred to nitrocellulose membranes, which were blocked, washed, and incubated for 1 hour with antibodies against TIMP-1 (1:250 dilution), collagenase-1 (1:250 dilution in Tris-buffered saline), or stromelysin-1 (1:500 dilution). The membranes were then washed, incubated with horseradish peroxidase-conjugated goat anti-rabbit antibody (1:1000 dilution), and washed again. Bands were revealed by incubation with enhanced chemiluminescence reagent and exposure to radiographic film. The bands for TIMP-1 western blots were quantified by videodensitometry as described [22]. Conditioned medium from pubic symphysis and articular cartilage explants was similarly subjected to western blot analysis for collagenase-1 and stromelysin-1.

### Substrate zymography

Enzyme activities were quantified by substrate zymography of conditioned media from 32 hemisections (mean wet weight 13 ± 9 mg). The samples were standardized by total protein and subjected to SDS–polyacrylamide-gel electrophoresis with 10% gels containing 2 mg/ml gelatin or casein at 15°C as described [22]. The gels were washed in 2.5% Triton X-100 for 30 min with one change of wash buffer, incubated at 37°C for 60–72 hours in incubation buffer (50 mM Tris-HCl buffer pH 8, 5 mM CaCl_2_, 0.02% NaN_3_), stained with 5% Coomassie blue, and destained in 10% acetic acid and 40% methanol until proteinase bands were clearly visible. Images of the gels were captured with a charge-coupled device camera and NIH image software. The levels of 53/58 kDa gelatinolytic and 51/54 kDa caseinolytic enzymes and their low-molecular-mass activated forms were quantified by videodensitometry [22]. The substrate zymograms rather than western blots were used to quantify hormone-mediated increases in proteinase levels because zymograms are more sensitive, often display both pro-forms and active forms of proteinases, show a greater linear range of densitometric values and have good reproducibility that together enable a reliable quantification of the enzymes from these gels [23-25]. In addition, gelatin zymograms selectively detect proteinase activity at 53/58 kDa and at 43 kDa attributable primarily to collagenase rather than stromelysin because gelatin is a poor substrate for stromelysin [25,26].

### Histochemical staining and quantification of GAGs

To assess changes in GAG levels, the discs were washed three times in PBS, frozen in OCT compound, and sectioned with a cryostat. The section were defrosted for 30 min, fixed for 10 min in methanol, air-dried for 15 min, stained with 1% Fast Green solution for 3 min, placed in 1% acetic acid for 1 min, stained with 2% Safranin-O for 2 min, dehydrated through successive ethanol and xylene washes, and mounted with coverslips. Ten sections of each hemisection were analyzed by an examiner blinded to the hormone treatment. The stained discs were videodigitized and analyzed with a software program that automatically outlined the total and Safranin-O-stained areas with threshold settings (Photoshop 4.0; Adobe, San Jose, California, USA). These areas were then quantified with NIH Image 1.62, and the percentage of disc staining positive for GAGs was calculated from the ratio of the stained area to the total area in each section. The average of the 10 values for each half disc was used for analysis.

### Determination of GAG synthesis by ^35^S radiolabeling

To quantify GAG biosynthesis, 32 disc hemisections (mean weight 14 ± 4 mg) were incubated at 37°C for 6 hours in 1 ml of phenol-free and serum-free medium with or without hormones and 165 kBq (0.0044 mCi) of ^35^S as described [27]. The discs were washed three times with medium containing 1 mg/ml sodium sulfate and digested for 24 hours with 20 U/ml papain. The digest (500 μl) was incubated for 30 min with 100 μl of 5% cetyl pyridiuium chloride in 0.3 M potassium chloride at room temperature (20–22°C) to precipitate GAGs. After centrifugation (3000 *g *for 20 min), the supernatant was removed and the precipitate was dissolved in 600 μl of concentrated formic acid by heating to 70°C for 10 min. Aliquots (20 μl) of this solution were added to 3 ml of scintillation fluid and subjected to liquid scintillation counting. The radioactivity (counts/min) was standardized to the total dry disc weight.

### Quantification of GAGs and collagen

Each disc hemisection was digested in 600 μl of 3 mg/ml pepsin in 0.05 M acetic acid and incubated at 37°C for 18–20 hours in a dry bath. DMMB binding assays for GAGs, and Sircol assays for collagen content, were performed in triplicate on 24 disc hemisections. The DMMB reagent was prepared as described [28]. Pepsin digests (200 μl) from each treatment group (GM6001 or analog control) were mixed with 1 ml of DMMB reagent, and absorbance at 525 nm was determined with a spectrophotometer. The GAG concentration (μg/ml) was determined by comparing the absorbance of the sample against a standard curve prepared from bovine chondroitin sulfate A, and the disc GAG content was standardized to the total dry tissue weight.

For the collagen assay, 200 μl of pepsin digest was mixed with 1 ml of Sircol dye reagent, incubated for 30 min at room temperature, and centrifuged at 10,000 *g *to separate the unbound dye from the collagen-bound dye. After removal of the unbound dye, 1 ml of the alkali reagent was added to the collagen–dye complex and vortex-mixed to dissolve the collagen-bound dye completely. Aliquots (200 μl) were transferred to the 96-well plates, and absorbance at 550 nm was determined with a microtiter plate reader (Molecular Devices, Sunnyvale, California, USA). The collagen concentration (μg/ml) was determined against a collagen standard curve, and the disc collagen content was standardized to the total disc dry weight.

### Quantification of collagenase activity

Collagenase activity in conditioned medium from discs cultured with GM6001 or control analog was assessed by FITC–collagen assay. A 96-well plate was coated with FITC–collagen (10 μg per well) overnight at 4°C and washed twice with PBS. Disc-conditioned medium (100 μl) was added to the wells, and the plate was incubated at 35°C for 1 hour. As a reference, 100 μl of blank medium containing 3000 ng of bacterial collagenase was added to one set of wells for complete digestion of FITC–collagen. After incubation, 90 μl from each well was transferred to another 96-well plate, and the fluorescence intensity of degraded FITC–collagen products was determined with a microplate spectrofluorometer (Spectramax Gemini XS; Molecular Devices) with excitation at 494 nm and emission at 518 nm. The data were converted to relative fluorescence units of collagenase activity as described by the manufacturer and standardized to the dry weight of each half disc. The fold differences in collagenase activity in medium from control and hormone-treated discs were determined for each experiment. All assays were performed in duplicate.

### Statistical analysis

Because of inherent variability in matrix content and proteinase activity in discs from different rabbits, three disc hemisections from each rabbit were treated with hormones and one served as control. MMP levels and the GAG and collagen content in each hormone-treated disc hemisection were standardized to the values of the control hemisection within each animal and the fold changes were plotted as histograms. The statistical significance of differences was determined by single-factorial analysis of variance (ANOVA). Intergroup differences were analyzed by Fisher's multiple comparisons test; *P *< 0.05 was considered statistically significant. Values are expressed as means ± SD.

## Results

### Relaxin and β-estradiol induce collagenase-1 and stromelysin-1 in TMJ disc explants

**Figure 1:**
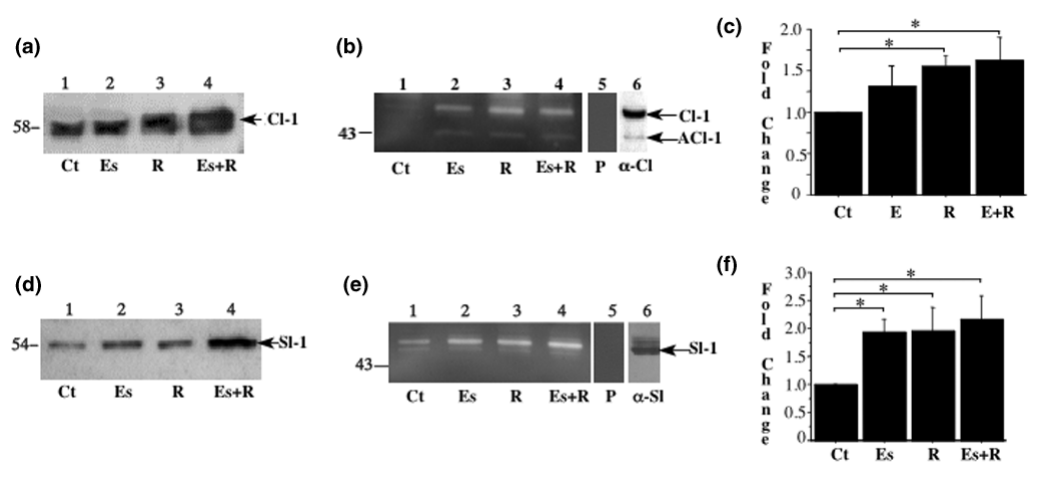
Relaxin induces collagenase-1 and stromelysin-1 in fibrocartilaginous explants from temporomandibular joint. Disc hemisections were exposed for 48 hours to basal control medium (Ct), β-estradiol (Es, 20 ng/ml), or relaxin (R, 0.1 ng/ml) or to β-estradiol plus relaxin (Es+R). Conditioned medium, standardized by tissue weight, was subjected to SDS–polyacrylamide-gel electrophoresis and transferred to membranes for western immunoblots for collagenase-1 **(a) **or stromelysin-1 **(d) **or assayed in gels containing gelatin **(b) **or α-casein **(e)**. Images of the substrate gels were digitized, and the 53/58 kDa and 43 kDa gelatinase activities (collagenase and active collagenase, respectively) **(c) **and the 51/54 kDa caseinolytic activity (stromelysin) **(f) **were quantified by videodensitometry. The samples used in lane 6 of panels (b) and (e) are positive controls for collagenase-1 and stromelysin-1. P, gels incubated in buffer containing the metalloproteinase inhibitor 1,10-phenanthroline; Cl-1, collagenase-1; ACl-1, active collagenase-1; Sl-1, stromelysin-1; α-Cl, anti-collagenase-1 antibody; α-Sl, anti-stromelysin-1 antibody. * *P *< 0.05.

Explanted discs constitutively expressed collagenase-1 (MMP-1) (Fig. 1a, lane 1), and the expression of this proteinase was increased by exposure to relaxin alone or to β-estradiol plus relaxin (Fig. 1a, lanes 3 and 4). Gelatin substrate zymograms confirmed the induction of 53/58 kDa proteinase by these hormones and, because this assay is more sensitive than western blots, showed an additional 43 kDa gelatinolytic enzyme (Fig. 1b). Because the gelatinolytic enzymes were inhibited by 1,10-phenanthroline (Fig. 1b, lane 5), these proteinases were characterized as MMPs, most probably procollagenase-1 and active collagenase-1. Western blots with conditioned medium from a disc explant exposed to relaxin showed that the 53/58 kDa and 43 kDa activities corresponded to procollagenase-1 and collagenase-1, respectively (Fig. 1b, lane 6). Proteinase expression was about 1.7-fold higher in relaxin-treated and β-estradiol plus relaxin-treated discs than in control cultures (*P *< 0.05) and was not potentiated by β-estradiol (Fig. 1c).

Because the expression of stromelysin and collagenase is often coordinately regulated [29], we assessed stromelysin expression. Western blots showed that all three hormone treatments induced stromelysin-1 (MMP-3) (Fig. 1d). Casein substrate zymograms demonstrated a 51/54 kDa caseinolytic proteinase (Fig. 1e, lanes 1–4) that was inhibited by 1,10-phenanthroline (Fig. 1e, lane 5), indicating a metalloprotease. This characterization was confirmed by western blotting (Fig. 1e, lane 6). Proteinase expression in relaxin-treated cultures was double that in control cultures (*P *< 0.05) and was not potentiated by β-estradiol.

### Relaxin induces collagenase-1 and stromelysin-1 in fibrocartilage but not in articular cartilage

**Figure 2:**
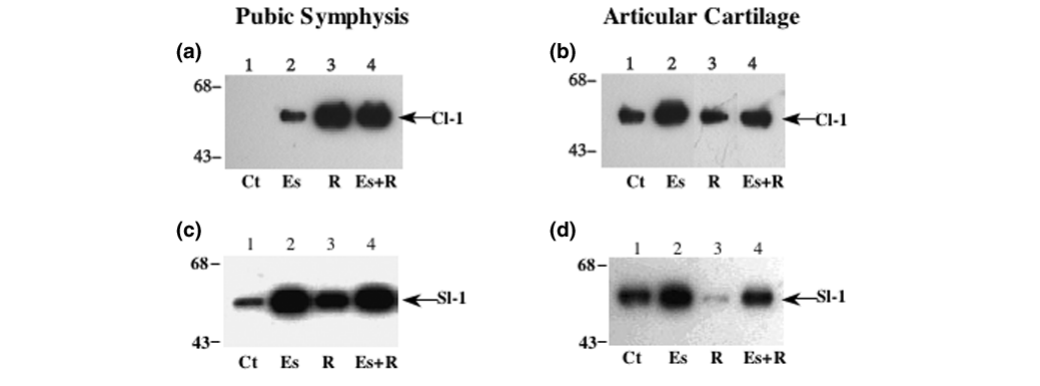
Relaxin induces collagenase-1 and stromelysin-1 in pubic symphysis fibrocartilage but not in articular cartilage. Pubic symphysis fibrocartilage or knee articular cartilage explants were exposed for 48 hours to basal control medium (Ct), β-estradiol (Es, 20 ng/ml), or relaxin (R, 0.1 ng/ml) or to β-estradiol plus relaxin (Es+R). Conditioned medium, standardized by tissue weight, was subjected to western blotting for collagenase-1 **(a, b) **or stromelysin-1 **(c, d)**. Cl-1, collagenase-1; Sl-1, stromelysin-1.

In pubic symphysis fibrocartilage, which is a known target site for β-estradiol and relaxin, β-estradiol caused slight increases in collagenase-1, while relaxin alone or in combination with β-estradiol induced a substantially greater expression of collagenase-1 relative to untreated discs (Fig. 2a). Relaxin also increased stromelysin-1 levels in pubic symphysis fibrocartilage. However, β-estradiol alone or in conjunction with relaxin produced substantially greater increases in stromelysin-1 levels than relaxin alone. In knee articular cartilage, although β-estradiol induced collagenase-1 and stromelysin-1, neither relaxin nor β-estradiol plus relaxin increased the expression of these proteinases over control levels (Fig. 2b,2d). Indeed, relaxin alone seemed to inhibit stromelysin-1 expression in articular cartilage.

### Loss of GAGs parallels the induction of MMPs by relaxin but not by β-estradiol

**Figure 3:**
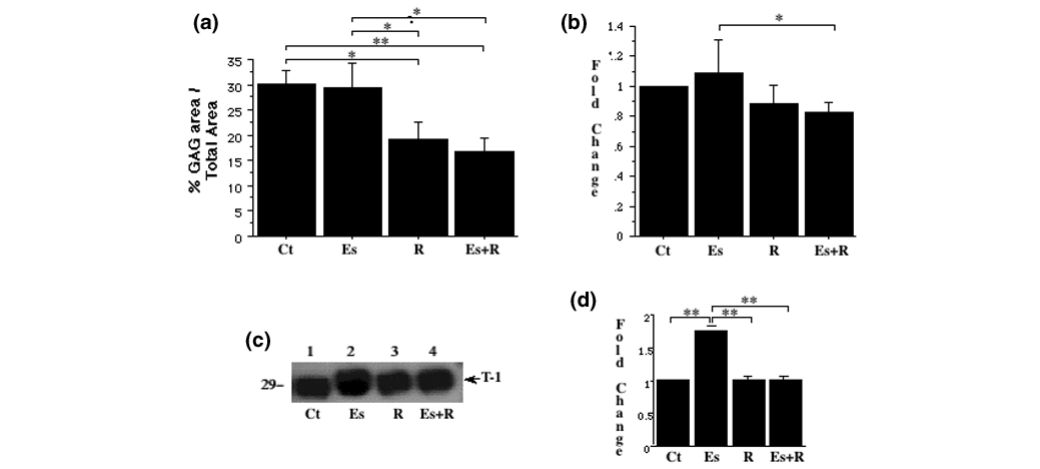
Induction of matrix metalloproteinases by relaxin but not by estrogen is accompanied by loss of glycosaminoglycans (GAGs). **(a) **Disc explants were cultured for 48 hours in basal control medium (Ct), β-estradiol (Es, 20 ng/ml), or relaxin (R, 0.1 ng/ml) or in β-estradiol plus relaxin (Es+R), then sectioned and stained with Safranin-O for GAGs. The percentage area staining positive for GAGs was determined histomorphometrically and plotted. Values are means ± SD. **(b) **Hormone-mediated changes in GAG synthesis were assessed by ^35^S-labeling of fibrocartilaginous disc explants. The explants were washed and digested with papain, and the radioactivity was measured. Fold changes (means ± SD) in ^35^S incorporated into the explants incubated with hormones relative to that in control discs were determined and plotted. **(c) **To evaluate the modulation of tissue inhibitor of metalloproteinases-1 (TIMP-1) by hormones, the conditioned medium, standardized dry tissue weight (mg), was resolved electrophoretically and transferred to nitrocellulose membranes, and the membranes were probed with anti-TIMP-1 antibody. **(d) **The bands were quantified by videodensitometry, and the fold induction (mean ± SD) of TIMP-1 by various hormone treatments relative to untreated control explants was plotted. T-1, TIMP-1. * *P *< 0.05, ** *P *< 0.01 by Fisher's test.

Because all hormone treatments induced stromelysin-1 expression in explanted discs, we assessed the level of a known substrate, proteoglycans, in Safranin-O-stained sections. The GAG-positive area was larger in control discs (30.1 ± 2.8% of total disc area) and discs treated with β-estradiol (29.7 ± 4.7%) than in those treated with relaxin (19.2 ± 3.3%) or β-estradiol plus relaxin (16.9 ± 2.7%) (Fig. 3a). These findings reflect statistically significant differences (*P *< 0.01, ANOVA) in GAG staining between control discs and those treated with relaxin (*P *< 0.05, Fisher's test) or β-estradiol plus relaxin (*P *< 0.01). Similarly, the GAG-positive area was significantly smaller (*P *< 0.04, ANOVA) in discs treated with relaxin (*P *< 0.05, Fisher's test) or β-estradiol plus relaxin (*P *< 0.05) than in those treated with β-estradiol alone.

### β-Estradiol induces TIMP-1

To determine why GAG loss did not increase in parallel with stromelysin expression in explants treated with β-estradiol alone, we assessed GAG synthesis and TIMP-1 expression. Except for a significantly lower GAG synthesis in discs exposed to β-estradiol plus relaxin than in those exposed to β-estradiol alone (*P *< 0.05), differences between the other groups were not significant (Fig. 3b). β-Estradiol caused a significant (*P *< 0.01) twofold induction in TIMP-1 expression over controls (Fig. 3c,3d). However, neither relaxin alone nor β-estradiol plus relaxin modulated any changes in TIMP-1 expression in the disc explants.

### Inhibition of MMP activity prevents relaxin-mediated loss of GAGs

**Figure 4:**
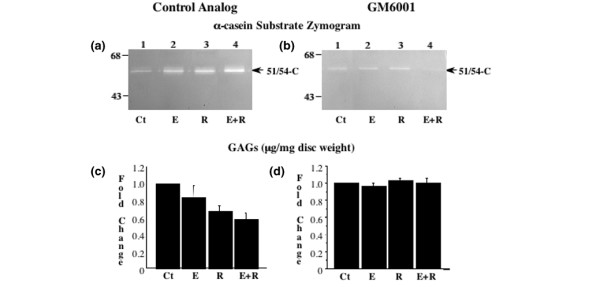
Inhibition of matrix metalloproteinase (MMP) activity prevents relaxin-mediated loss of glycosaminoglycans (GAGs). Conditioned medium from disc hemisections incubated with β-estradiol (Es), relaxin (R), or β-estradiol plus relaxin (Es+R) in the presence of the MMP inhibitor GM6001 or its control analog was assayed by casein substrate zymograms **(a, b)**. Disc digests from these experiments were assayed for GAGs with the 1,9-dimethylmethylene blue assay, and the results were standardized to tissue dry weight (mg). Fold changes in GAG concentration (mean ± SD) were calculated and plotted **(c, d)**. The untreated control (Ct) discs used in all experiments were exposed to control analog only. * *P *< 0.05, ** *P *< 0.01, *** *P *< 0.001 by Fisher's test.

To establish an association between the increased MMP activity and the loss of GAGs in explants treated with relaxin or β-estradiol plus relaxin, we cultured the explants with the MMP inhibitor GM6001 or its control analog. Western blot analysis showed a higher expression of stromelysin-1 in hormone-treated than untreated disc explants in the presence of GM6001 or its control analog (data not shown). However, zymography showed increased 51/54 kDa caseinolytic activity (stromelysin-1) only in hormone-treated explants incubated with the control analog (Fig. 4a), and not in those incubated with GM6001 (Fig. 4b).

DMMB assays showed that hormone treatments in the presence of control analog decreased the GAG content (*P *< 0.0001, ANOVA), which was 30% lower in relaxin-treated explants (*P *< 0.001, Fisher's test) and 40% lower in those treated with β-estradiol plus relaxin (*P *< 0.001) than in untreated explants (Fig. 4c). Similarly, the GAG content was lower (*P *< 0.0001, ANOVA) in discs treated with relaxin (*P *< 0.05, Fisher's test) or β-estradiol plus relaxin (*P *< 0.001) than in those treated with β-estradiol alone. In the presence of GM6001, however, hormone treatments did not affect the GAG content (Fig. 4d).

### Relaxin-induced collagenase activity contributes to loss of disc collagen

**Figure 5:**
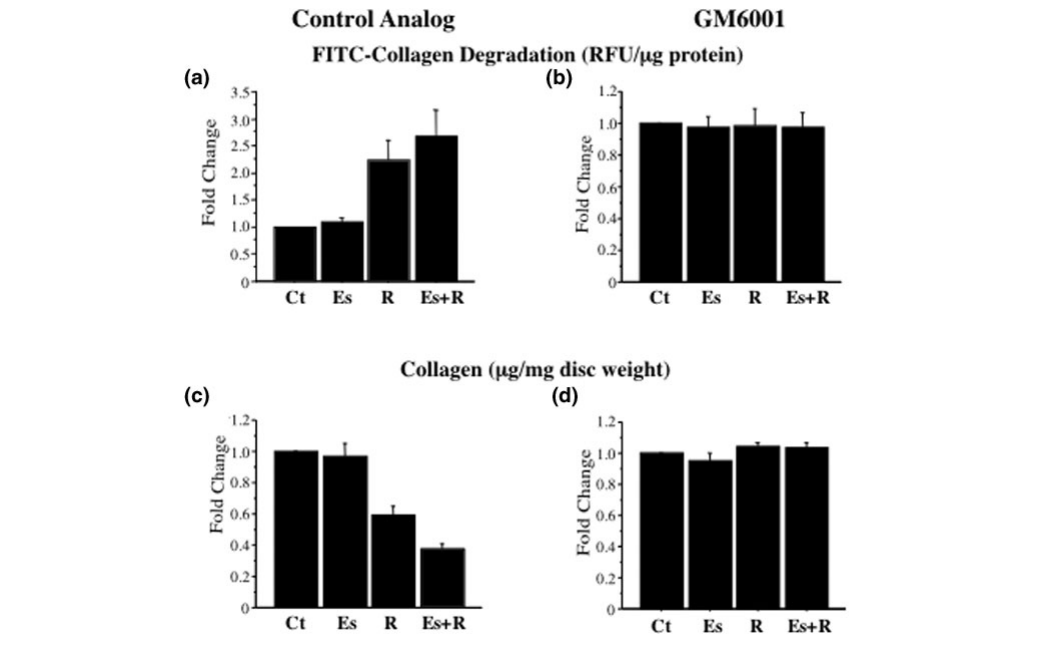
Relaxin-induced collagenase activity contributes to loss of disc collagen. Conditioned medium from disc incubated with control medium (Ct), β-estradiol (Es), relaxin (R), or β-estradiol plus relaxin (Es+R) in the presence of the matrix metalloproteinase inhibitor GM6001 or its control analog was subjected to fluorescein isothiocyanate-labelled collagen degradation assay. The collagenase activity (relative fluorescence units [RFU]/ml) was standardized by the dry weight of the tissue (mg), and fold changes (means ± SD) were plotted **(a, b)**. Disc digests from these experiments were assayed for collagen with the Sircol assay, and the results were standardized to tissue dry weight (mg). Fold changes in collagen concentration (means ± SD) were calculated and plotted **(c, d)**. The untreated control (Ct) discs used in all experiments were exposed to control analog only. ** *P *< 0.01, *** *P *< 0.0001 by Fisher's test.

All three hormone treatments increased the expression of procollagenase-1 in the presence of GM6001 or its control analog similarly to that shown in Fig. 1a,1b,1c. However, as shown by FITC-collagen degradation assays, collagenase activity was significantly increased only by relaxin or β-estradiol plus relaxin in the presence of the control analog (Fig. 5a). In discs incubated with GM6001, hormone-induced collagenase activity was inhibited to control levels (Fig. 5b). Conversely, Sircol assays showed the collagen content was significantly decreased (*P *< 0.0001, ANOVA) only in the presence of the control analog and only by relaxin (40% of control and β-estradiol alone; *P *< 0.0001, Fisher's test) or β-estradiol plus relaxin (60% versus control and β-estradiol alone; *P *< 0.0001) (Fig. 5c). In the presence of GM6001, hormone treatments did not affect collagen content (Fig. 5d).

## Discussion

This study shows that relaxin induced the expression of collagenase-1 and stromelysin-1 in rabbit TMJ disc explants, accompanied by a loss of GAGs and collagen, but did not affect GAG synthesis. In explants cultured with the MMP inhibitor GM6001, collagenase-1 and stromelysin-1 activities in hormone-treated discs were inhibited to baseline levels, and collagen and GAG content were maintained at control levels. These findings show that relaxin has degradative effects on nonreproductive synovial joint fibrocartilaginous tissue and provide evidence that increases in MMP activity mediated by relaxin and β-estradiol plus relaxin contribute directly to the loss of disc collagen and GAGs. The lack of effect on GAG synthesis further validates the importance of the degradative component of the remodeling cycle in relaxin's modulation of matrix loss in fibrocartilage.

Because the MMP inhibitor used in our studies is not specific for collagenase-1 and stromelysin-1, the hormone-induced loss of collagen and GAGs cannot be specifically linked to those two proteinases. Rather, our findings implicate MMPs in general in this response. However, because GM6001 has a low dissociation constant for both collagenase-1 and stromelysin-1 [30], and their induction by relaxin was accompanied by a loss of their matrix substrates, collagenase-1 and stromelysin-1 are probably involved in the relaxin-mediated loss of collagen and GAGs, respectively.

In contrast to the results obtained with relaxin and β-estradiol plus relaxin, the induction of collagenase-1 and stromelysin-1 by β-estradiol alone was not accompanied by changes in GAG or collagen content within the disc. How can we explain this apparent discrepancy? β-Estradiol had little effect on GAG synthesis, as measured by ^35^S incorporation, but it produced a statistically significant increase in TIMP-1 expression that could have counteracted any increases in degradative activity due to increased expression of collagenase-1 and stromelysin-1. Indeed, the results of the collagen degradation assay lend credence to this hypothesis. These findings imply that relaxin and β-estradiol selectively contribute to the degeneration of fibrocartilaginous tissue by differentially modulating MMP expression, matrix synthesis, and net matrix content.

The potential similarities in the responsiveness of TMJ fibrocartilaginous explants and the pubic symphysis fibrocartilage to relaxin are reflected not only by the relaxin's induction of collagenase but also by the comparable loss of collagen on the exposure of these tissues to the hormone [4,9]. Thus, the extent of collagen loss in fibrocartilaginous disc explants exposed to relaxin (40%) or β-estradiol plus relaxin (60%) was similar to that in the pubic symphysis of unprimed and β-estradiol-primed ovariectomized nonpregnant rats (64 ± 4% and 68 ± 6%, respectively) [4]. Similarly, in pregnant ovariectomized rats, relaxin decreased collagen to 39% of the levels in nonpregnant animals [9]. Additionally, β-estradiol alone had minimal effects on the collagen content of the fibrocartilaginous TMJ disc, which is also similar to observations on the pubic symphysis [4,9]. Thus, relaxin with or without β-estradiol, but not β-estradiol alone, has a potent effect on the amount of collagen in fibrocartilaginous tissues from different sites, including the pubic symphysis and synovial joints. These findings also suggest that in fibrocartilaginous tissues, including the TMJ disc and possibly the pubic symphysis, relaxin decreases collagen and GAG content primarily by inducing MMP expression.

The response of articular cartilage to relaxin or β-estradiol plus relaxin was substantially different from that of the TMJ disc and pubic symphysis fibrocartilages. Although the reasons for these differences remain to be determined, it is well accepted that articular cartilage is a cartilaginous tissue containing chondrocytic cells, whereas fibrocartilage is a heterogenous tissue composed of cartilage and fibrous tissue that contains cells of fibroblastic, chondrocytic, and fibrochondocytic phenotypes. It is plausible that of these cells, the fibroblastic and/or fibrochondrocytic cells found in fibrocartilage, rather than the chondrocytic cells, are those that produce the observed responses to relaxin and β-estradiol plus relaxin. Indeed, previous findings on both dermal fibroblasts showing a potent induction of MMP-1 [18] and on articular chondrocytes that show minimal modulation of total collagen synthesis by relaxin [31] lend credence to this hypothesis. Additional studies are indicated to address the mechanistic basis for the differences in responsiveness of fibrocartilaginous versus cartilaginous cells to relaxin.

Our findings are consistent with emerging data suggesting that the mechanisms for the loss of matrix macromolecules caused by relaxin are tissue-specific [32]. Thus, for example whereas relaxin increases collagenase-1 expression in TMJ disc and pubic symphyseal [6] fibrocartilages, it had minimal effects on its expression in articular cartilage explants. In monolayer articular or multilayer growth plate rabbit chondrocytes, relaxin produces no net change in collagen synthesis and no alterations in type II collagen mRNA levels, but increases the expression of types I and III collagen mRNA, thereby amplifying the dedifferentiation process [31]. In contrast, relaxin downregulates collagen expression by up to 40% and induces collagenase expression in cultured dermal fibroblasts [18]. As in our study, relaxin increases collagenase activity in human cervical stromal cells; however, in contrast to our findings, it also increases GAG synthesis [15,16].

MMPs contribute substantially to tissue degeneration in inflammatory joint diseases, including rheumatoid arthritis and osteoarthritis [33-35]. Our findings show that relaxin directly modulates MMP expression and probably causes matrix loss in fibrocartilaginous tissues from a synovial joint. Although the effects of relaxin on loss of matrix macromolecules, particularly collagen, have been demonstrated in the fibrocartilaginous pubic symphysis [4-7], this is the first study to demonstrate a similar targeting of fibrocartilaginous tissues from the synovial TMJ, and may implicate this hormone in the pathogenesis of TMJ disease in a subset of women with these disorders. Because even subtle alterations in collagen and GAG composition can affect the structural properties and the ability of joint tissues to function normally, this modulation of MMPs and resulting matrix loss in the fibrocartilaginous TMJ disc by relaxin might explain the distinct age and gender distribution of TMJ diseases. Furthermore, these findings have potential physiologic relevance because the induction of collagenase-1 and stromelysin-1 and the loss of collagen and GAGs occurred at concentrations of relaxin found systemically in cycling women [36-38]. Although the ability of systemic relaxin to access the TMJ and reach the avascular disc remains to be determined, our recent findings *in vivo *showing relaxin-mediated decreases in GAG concentration in the TMJ discs of ovariectomized rabbits suggest that this systemic hormone can indeed access the TMJ disc and contribute to its degradation [39].

## Conclusions

Relaxin causes the targeted induction of collagenase-1 and stromelysin-1 in synovial joint and pubic symphysis fibrocartilages but not in articular cartilage. This induction of MMPs in joint fibrocartilage is accompanied by a loss of collagen and GAGs that is prevented by an MMP inhibitor, suggesting a link between relaxin, MMPs, and matrix degradation. These studies provide the first evidence that relaxin contributes to the degradative remodeling of joint fibrocartilage and that there is an association between relaxin-induced MMPs and matrix loss; they also suggest a potential mechanism of action of relaxin in contributing to TMJ diseases in a subset of women with these disorders.

## Competing interests

The author(s) declare that they have no competing interests.

## Authors' contributions

TN performed all experiments, assays and analysis in which MMP inhibitors were used. TTD performed all experiments to characterize the changes in MMPs and GAGs in joint fibrocartilage in response to relaxin and β-estradiol. GH and QZ characterized the responses of the pubic symphysis fibrocartilage and articular cartilage to the hormones. MS retrieved tissues from animals and assisted in several MMP assays. SK conceived the study, participated in its design and coordination, supervised the statistical analysis, and wrote the manuscript. All authors read and approved the final manuscript.

## Abbreviations

ANOVA = analysis of variance; DMMB = 1,9-dimethylmethylene blue; GAG = glycosaminoglycan; FITC = fluorescin isothycyanate; MMP = matrix metalloproteinase; PBS = phosphate-buffered saline; TIMP = tissue inhibitor of metalloproteinase; TMJ = temporomandibular joint.
